# The Diversity of Nitrogen-Cycling Microbial Genes in a Waste Stabilization Pond Reveals Changes over Space and Time that Is Uncoupled to Changing Nitrogen Chemistry

**DOI:** 10.1007/s00248-020-01639-x

**Published:** 2020-11-10

**Authors:** A. Rose, A. Padovan, K. Christian, J. van de Kamp, M. Kaestli, S. Tsoukalis, L. Bodrossy, K. Gibb

**Affiliations:** 1 Research Institute for the Environment and Livelihoods, Charles Darwin University, Darwin, Northern Territory 0909 Australia; 2 CSIRO Oceans and Atmosphere, Hobart, Tasmania 7004 Australia; 3 PowerWater Corporation, Darwin, Northern Territory 0820 Australia

**Keywords:** Bacteria, Archaea, Functional gene array, Nitrogen cycle, Nutrients, Wastewater stabilization ponds

## Abstract

Nitrogen removal is an important process for wastewater ponds prior to effluent release. Bacteria and archaea can drive nitrogen removal if they possess the genes required to metabolize nitrogen. In the tropical savanna of northern Australia, we identified the previously unresolved microbial communities responsible for nitrogen cycling in a multi-pond wastewater stabilization system by measuring genomic DNA and cDNA for the following: *nifH* (nitrogen fixation); *nosZ* (denitrification); *hzsA* (anammox); archaeal *AamoA* and bacterial *BamoA* (ammonia oxidation); *nxrB* (nitrite oxidation); and *nrfA* (dissimilatory NO_3_ reduction to NH_3_). By collecting 160 DNA and 40 cDNA wastewater samples and measuring nitrogen (N)-cycling genes using a functional gene array, we found that genes from all steps of the N cycle were present and, except for *nxrB*, were also expressed. As expected, N-cycling communities showed daily, seasonal, and yearly shifts. However, contrary to our prediction, probes from most functional groups, excluding *nosZ* and *AamoA*, were different between ponds. Further, different genes that perform the same N-cycling role sometimes had different trends over space and time, resulting in only weak correlations between the different functional communities. Although N-cycling communities were correlated with wastewater nitrogen levels and physico-chemistry, the relationship was not strong enough to reliably predict the presence or diversity of N-cycling microbes. The complex and dynamic response of these genes to other functional groups and the changing physico-chemical environment provides insight into why altering wastewater pond conditions can result an abundance of some gene variants while others are lost.

**Supplementary Information:**

The online version contains supplementary material available at 10.1007/s00248-020-01639-x.

## Introduction

Over half a decade ago, nitrogen (N) removal in wastewater stabilization pond (WSP) systems was considered unpredictable. Along with pathogen removal, it is critical for WSPs to efficiently remove N from wastewater to prevent nutrient pollution in the receiving waterbodies. Consequently, if unreliable, WSP N removal can be expensive if pond effluent requires further treatment before it is discharged into the environment.

How and where N is lost in a multi-pond wastewater system is still debated. Ammonia volatilisation and N sedimentation into the pond sludge are considered by some to be the two main removal pathways [1, 2]. Thus, it is assumed that most N is removed in the first ponds because they enhance volatilisation and settlement into the sludge. Ammonia volatilisation is accelerated in these initial ponds because they receive highly concentrated organic N from the raw influent. The organic N readily mineralises and converts to ammonia which then volatises to N_2_ gas and emits into the atmosphere. The rate of the ammonia volatilisation depends on the water’s ammonia gas concentration, temperature, pH, and pond depth [3]. However, the importance of ammonia volatilisation has come into question with studies on wastewater systems finding N removal by volatilisation insignificant [4, 5]. Instead, these studies suggested that N is lost through simultaneous nitrification-denitrification in a process called the nitrogen cycle (N cycle). The coupled nitrification-denitrification process requires pond water to have both high and low oxygen environments. However, even if ponds appear to only have one of these oxygen environments, new evidence suggests that both environments can co-occur and allow coupled nitrification-denitrification because of the existence of micro-domains in most wastewater ponds. Micro-domains can exist in WSPs because of the symbiotic relationship between photosynthetic algae and aerobic bacteria that can create high oxygenated micro-domains for nitrification during the day [6]. At the same time, drifting sludge mats can consume oxygen directly underneath, thus promoting denitrification [7]. Therefore, in light of the recent N removal work in WSPs, the focus has broadened beyond the role of ammonia volatilisation and N sedimentation to include the entire nitrogen cycle.

**Fig. 1:**
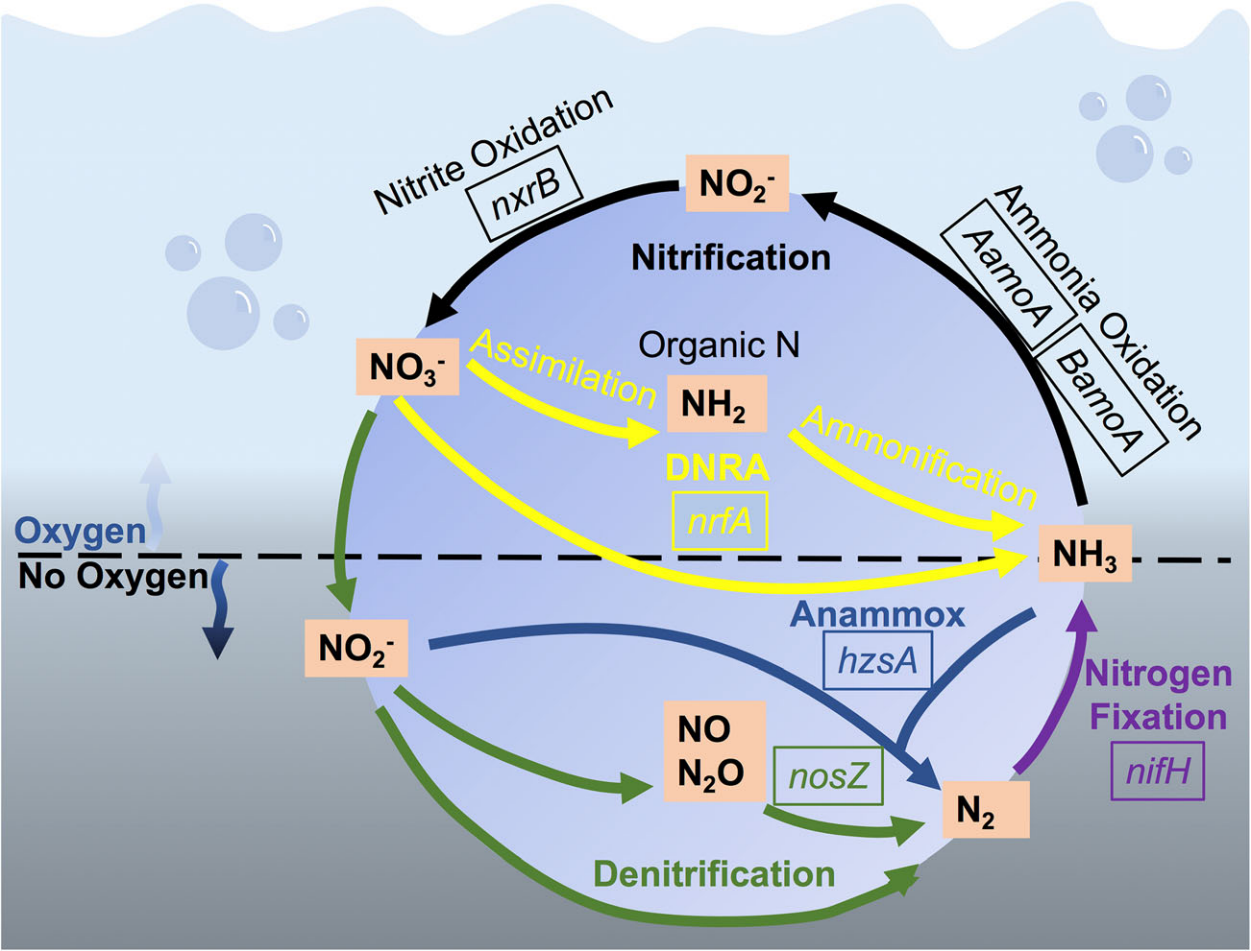
Nitrogen cycle activity in the WSP adapted from the Bernhard [8] schematic. Arrows indicate direction of reaction. Genes associated with nitrogen-cycling pathways include as follows: *nrfA*, DNRA (dissimilatory NO_3_ reduction to NH_3_); *nosZ*, denitrification; *hszA*, anammox; *nifH*, nitrogen fixation; *AamoA* and *BamoA*, ammonia oxidation; *nxrB*, nitrite oxidation. The dotted line indicates the interface between the high and low oxygen environments needed for each pathway

Bacteria and archaea drive the nitrification-denitrification processes. Therefore, to understand N loss from wastewater, it is critical to identify the N-cycling genes that are present and active in the system. For example, the nitrification pathway occurs when oxygen is present and requires the presence of different microbes with the following genes: *AamoA* (archaea) or *BamoA* (bacteria) for ammonia oxidation; *nxrB* for nitrite oxidation; while *nrfA* encodes the enzyme for dissimilatory nitrate reduction to ammonia (Fig. 1). Conversely, the denitrification, anammox, and nitrogen fixation pathways occur in the absence of oxygen and require the genes *nosZ* for denitrification of NO/N_2_O to N_2_ gas, *hzsA* for anammox, and *nifH* for nitrogen fixation (Fig. 1). A functional gene array (FGA) is an ideal approach because it allows an efficient and targeted search for N-cycling microbes [9]. Because FGAs are a rapid and cost-effective method for detecting microbes and their functional genes from virtually any sample, they can be applied to a wide array of sample types [9, 10]. For example, FGA studies investigating nitrogen cycling associated with harmful cyanobacterial and dinoflagellate blooms in freshwater and marine environments showed that genes and bacteria driving N cycling were spatially and temporally dynamic [11, 12].

Because of a lack of understanding of the N-cycling communities, previous wastewater systems were developed without considering the key microbes involved in N treatment. Consideration of N-cycling groups was further confounded by the complicated relationship bacteria and archaea have with the surrounding physical environment and chemical substrates they use [9]. It is well established that the physical environment can influence N-cycling transformation pathways. For example, nitrification fails when the pH falls below 7.2 and temperature is not within 5–30 °C [3]. Similarly, the environment can also determine the abundance of different functional groups of N-cycling microbes. For example, ammonia oxidisers (*AamoA* and *BamoA*) are competitive under low oxygen conditions and low NH_4_^+^-N concentrations [9, 13–16]. Thus, it is not surprising that wastewater physico-chemistry and the N substrate concentration can influence the dominance of functional microbial groups.

It is well known that the climate and geographic location also influence the presence and activity of the N-cycling communities that drive N removal and transformation. Comparisons of numerous worldwide studies on N removal and transformation in WSPs show how the changing environmental conditions influence the N-cycling process and microbes involved [3, 17–21]. These studies show that shifting environmental conditions over space and time changed the N transformation along with the microbial community and diversity because N-cycling microbes were habitat specific [9]. Subsequently, for each WSP, it is important to take multiple measurements of the N-cycling community and water chemistry because treatment systems harbour different N-cycling communities and a single measurement in time does not capture temporal variation, which may confound WSP management decisions.

In this study, we used the novel nitrogen cycle FGA to identify the functional communities driving the N cycle in a wet-dry tropical WSP. We defined a functional community as a microbial gene that catalyses the same step in the N cycle. For example, *nosZ* genes belong to the denitrification community. The WSP has two distinct climatic conditions (wet and dry seasons) and daily fluctuation in dissolved oxygen (DO) levels from algal photosynthesis. We identified the influence of these factors on the N-cycling functional communities by measuring the N-cycling genes at yearly, seasonal, and daily intervals, including whether or not the genes were active. We hypothesised that each functional community would show small (daily) and large (season/yearly) temporal shifts in gene diversity in response to the changing environmental conditions. However, for each time point, we expected the communities to remain similar between the inlet and outlet of each pond and between the facultative and maturation ponds because of possible micro-domains that could facilitate coupled nitrification-denitrification throughout the system. We expected functional communities to increase in relative abundance and diversity with the rise in concentration of their complementary N substrate. We reasoned that if N substrate levels were in fact a surrogate for changes in N-cycling community diversity, we could predict WSP community patterns along a nutrient gradient. Understanding the N-cycling communities in a multi-pond system will allow operators to understand where and how N is removed in the tropical system and which microbial genes are involved. Consequently, operators can utilize this information to optimize existing systems or built new systems to efficiently remove N.

## Material and Methods

### Study Site

**Fig. 2:**
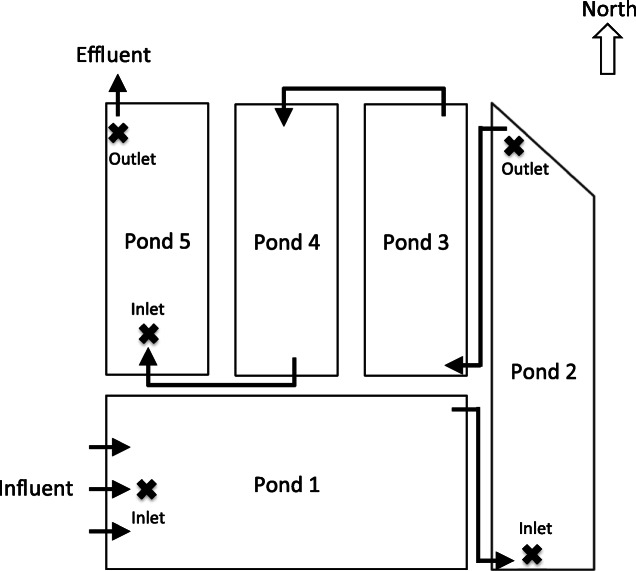
Schematic of the WSP showing sample locations (X) in ponds 1, 2, and 5 and flow direction. Schematic adapted from Rose et al. [22]

The WSP services approximately 50,000 customers in Darwin (NT, Australia) (12.4634° S 130.8456° E). The five-pond system comprises one facultative and four maturation ponds (Fig. 2). Raw influent enters the system through three inlets into the facultative pond. Effluent then feeds into a 4-pond maturation series for sanitation, before final release of treated water (Fig. 2). During the wet season (November–April), monsoonal rainfall results in “dilute” wastewater, with significant decreases in nutrient concentrations, while the opposite is true during the dry season (May–October), when evaporation is high.

### Wastewater Collection

In 2012 and 2013, wastewater samples (*n* = 160) were collected from the inlet of pond 1, and inlet and outlet of pond 2 and pond 5 on four occasions during the wet and dry seasons. For each field campaign, duplicate samples were collected from each site from the top 10 cm of the water column and bottom 10 cm in the morning (6 am–10 am) and again in the afternoon (1 pm–5 pm). To test and confirm the presence of N-cycling gene expression (cDNA), a subset of samples (*n* = 40) was collected from the surface waters in the afternoon. The following volumes were collected: 1 L for DNA and cDNA FGA analysis; 1 L for nutrients; 500 mL for biological oxygen demand (BOD); 250 mL for total organic carbon (TOC), total suspended solids (TSS)/total volatile solids (VSS); and 100 mL for alkalinity. All samples were placed on ice in the field, then kept at 4 °C until analyses were performed. In situ measurements of DO, temperature, conductivity, and pH were simultaneously recorded using the HYDROLAB**®** Quanta**®**.

### DNA and RNA Extraction, cDNA Preparation, and Processing of N Chemistry and Physico-Chemistry

Wastewater DNA and RNA extractions, cDNA synthesis (created with random hexamers), and N chemistry and wastewater physico-chemistry (TP, PO_4_^+^, BOD, TOC, TSS, VSS, and alkalinity) were processed using the same methods as outlined in Rose et al. [22].

### Functional Gene Microarray

High-throughput FGA was performed at the CSIRO Oceans and Atmosphere laboratory (Hobart, Tasmania, Australia) to assess the relative abundance and diversity of denitrification (*nosZ*), anammox (*hzsA*), nitrogen fixation (*nifH*), ammonia oxidation (*AamoA* and *BamoA*), nitrite oxidation (*nxrB*), and dissimilatory NO_3_ reduction to NH_3_ (*nrfA*) bacteria in WSP water samples (Fig. 1). Briefly, the FGA consists of a small solid substrate (glass microscope slide) to which a set of targeted oligonucleotide probes is attached. The functional genes of interest (*nosZ*, *hzsA*, *nifH*, *AamoA*, *BamoA*, *nxrB*, and *nrfA*) and the primers used for their amplification are listed in Supplementary Table 1. Amplification of partial N-cycle functional marker gene fragments was achieved via PCR using primers and cycling conditions shown in Supplementary Table 1. The *hzsA* fragment was amplified via a nested protocol [23]. PCR amplifications were carried out in 96-well plates, with 25 μL volumes, and contained 1× GoTaq mix (Promega), 40 nM of forward primer, 0.1 μL of 50 ng/μL molecular-grade BSA (Promega), and 10 ng environmental DNA or cDNA. Amplicons for both genomic DNA and cDNA were fluorescently labelled by in vitro transcription and labelled with Cy3-UTP, and hybridized on an array containing multiple probes for *nifH* (144), *nosZ* (182), *hzsA* (44), *AamoA* (60), *BamoA* (21), *nxrB* (21), and *nrfA* (182) covering multiple bacterial and archaeal clades (Supplementary Table 2). Signals were normalized to a spike control, set to 10,000. Detailed information about the development and methods of the FGA is provided in Supplementary Information [22].

### Statistical Analysis and Visualisation

Physico-chemical, N chemistry, and FGA data were analysed with PRIMER V7 PRIMER and PERMANOVA+ (Primer-E Ltd., Plymouth, UK), R© (The R Foundation for Statistical Computing, Vienna, Austria), RStudio Inc. (Delaware corporation, MA 02210), and Minitab® V6 Statistical Software. Physicochemical and N chemistry data were normalized and a resemblance matrix generated based on Euclidean distance, while FGA data was square-root transformed and a resemblance matrix generated based on the Bray-Curtis similarity. A permutational ANOVA (PERMANOVA) with 999 permutations was used to explore differences in FGA or physicochemical data between groups of samples. The PERMANOVA crossed design for both physicochemical and FGA DNA data (excluding cDNA) included 6 fixed factors or groups of samples: year (2 levels), season (2 levels), pond (3 levels), location (2 levels), time (2 levels), and depth (2 levels). A *P* value of <0.05 (2-sided) was considered significant. PermDISP (Primer-E Ltd., Plymouth, UK) was used to check for homogeneity of dispersions between groups. The N-cycling community structure and diversity of *nifH*, *nrfA*, *AamoA*, *BamoA*, *nxrB*, *nosZ*, and *hzsA* FGA DNA and cDNA data were visualized with heatmaps generated in RStudio Inc. and functional diversity measured using Shannon’s diversity. In addition to PERMANOVA, analysis comparisons of overall patterns for sites between groups (*nifH*, *AamoA, BamoA*, *nxrB*, *nrfA*, *nosZ*, and *hzsA*) were further explored using the 2nd-stage analysis in PRIMER and any significant differences between populations tested using Spearman’s coefficient in Minitab®. The relationship between *nifH*, *nrfA*, *AamoA, BamoA*, *nxrB*, *nosZ*, and *hzsA* communities with N chemistry concentration and physico-chemistry was explored using the DistLM analysis in PRIMER. For the diverse *nifH* and *nosZ* communities, indicator genes that were present in 90% of samples and drove the significant differences in the communities between the ponds and years (or seasons) were identified by IndVal in R and visualized using Cytoscape (Institute of Systems Biology, Seattle).

## Results

### WSP N-Cycling Gene Diversity (DNA)

**Fig. 3:**
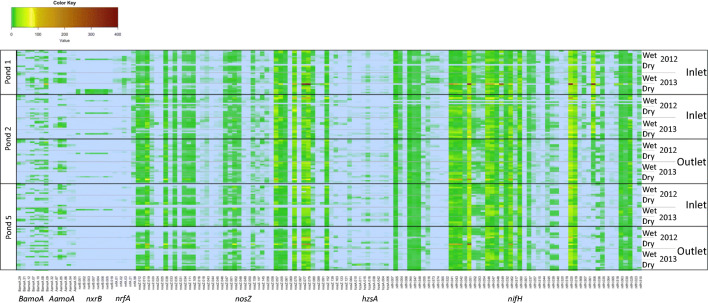
Heatmap of *BamoA*, *AamoA*, *nxrB*, *nrfA*, *nosZ*, *hzsA*, and *nifH* FGA DNA in ponds 1, 2, and 5. For clarity, a subset (out of total) of 7 (99) *BamoA*, 6 (60) *AamoA*, 8 (21) *nxrB*, 5 (138) *nrfA*, 47 (182) *nosZ*, 8 (42) *hzsA*, and 55 (144) *nifH* probes are shown in the Fig. A value of 100 means the signal was equal to that of the control probe (hyaBP60), whereas a value of 10 indicates that the signal was 10% of the control. Colour coding is indicated on the colour bar on top of heatmap. All sample values are shown (not averaged). See Supplementary Table 2 for probe label and taxon identification details and the FGA data_DNA supplementary excel for results values

We observed positive probe signals for representatives from all of the N-cycling functional communities associated with the nitrogen cycle that we tested, but not all the probes for each functional community hybridized to the DNA. The number of probes which hybridized compared to the total number of probes tested within each N-cycling functional community is as follows: *AamoA* (6/60), *BamoA* (7/99), *nxrB* (8/21), *nrfA* (5/138), *nosZ* (55/182), *hzsA* (8/44), and *nifH* (47/144) (Fig. 3). The diversity and relative abundance of the positive N-cycling probes changed over time (year, season, or time of day) and space (pond number or location) (Fig. 3 and Table 1). For example, all functional groups, except *nrfA*, had different positive probes between ponds, and *nrfA*, *nosZ*, and *nifH* probes differed between the inlet and outlet of the ponds. Further, despite the presence of some probes that were always detected at similar relative abundances irrespective of time and space, generally, positive *nifH* and *nosZ* probes were different at all macro- (year and season) and micro- (time of day) timescales (Fig. 3 and Table 1). However, signals for *AamoA* and *hzsA* probes differed on a yearly and daily basis, but not between seasons, while *nrfA* probes differed yearly and seasonally but did not change daily. *BamoA* probe signals only differed between seasons while *nxrB* only differed between years. Spearman’s ranked 2nd-stage analysis of the seven functional N-cycling communities showed weak correlations between community patterns over space and time (Supplementary Table 3). For example, with a *R*^2^ value of only 0.38, the *nosZ* and *nifH* communities showed the strongest correlation in their temporal and spatial patterns (see supplementary material for more details on the taxon identification for each N-cycling community).

**Table 1:** PERMANOVA tests for differences in the positive probe composition of N-cycling communities between year (2012 and 2013), season (wet and dry), pond (ponds 1, 2, and 5), pond location (inlet, middle, and outlet), time of day (6 am and 1 pm), and water depth (surface and benthic)

PERMANOVA factor	Pseudo-F (df)	ECV	*P* value	PermDISP *P* value
*AamoA* probes (> 997 unique permutations, residual ECV = 42)				
Year	53.7 (1)	38.2	0.001***	0.9
Season	1.2 (1)	2.1	0.3	0.03*
Pond	3.2 (2)	9.1	0.01**	0.7
Location	1.0 (1)	− 0.2	0.4	0.3
Time of day	6.0 (1)	11.7	0.002**	0.005**
Depth	0.2 (1)	− 4.7	0.9	0.8
Year × time	4.8 (1)	14.5	0.005**	0.008**
*BamoA* probes (> 996 unique permutations, residual ECV = 52)				
Year	1.8 (1)	5.9	0.1	0.3
Season	5.7 (1)	14.1	0.002**	0.3
Pond	2.6 (2)	9.6	0.02*	0.3
Location	0.4 (1)	−5.1	0.8	0.6
Time of day	0.6 (1)	−4.2	0.7	0.3
Depth	0.7 (1)	−3.6	0.6	0.5
Year × time	5.3 (1)	19.0	0.004**	0.005**
*nrfA* probes (> 997 unique permutations, residual ECV = 36.2)				
Year	3.9 (1)	7.7	0.02*	0.03*
Season	5.4 (1)	9.5	0.004**	0.4
Pond	40.4 (2)	32.7	0.001***	0.2
Location	13.0 (1)	15.6	0.001***	0.7
Time of day	0.1 (1)	−4.3	0.9	0.5
Depth	1.3 (1)	2.3	0.3	0.9
Year × season	9.8 (1)	19.0	0.002**	0.3
*nxrB* probes (> 997 unique permutations, residual ECV = 24.9)				
Year	6.1 (1)	7.0	0.009**	0.01**
Season	2.8 (1)	4.2	0.08	0.4
Pond	2.8 (2)	4.8	0.06	0.03*
Location	1.2 (1)	1.4	0.3	0.09
Time of day	0.9 (1)	−0.9	0.4	0.6
Depth	1.6 (1)	2.4	0.2	0.4
Year × depth	4.7 (1)	8.5	0.02*	0.04*
*hzsA* probes (> 997 unique permutations, residual ECV = 39.9)				
Year	4.4 (1)	9.2	0.02*	0.8
Season	1.4 (1)	3.2	0.2	0.01**
Pond	4.3 (2)	10.4	0.002**	0.05*
Location	0.7 (1)	−0.3	0.6	0.9
Time of day	10.2 (1)	15.1	0.001***	0.8
Depth	2.3 (1)	5.7	0.08	0.2
Season × time	15.2 (1)	19.5	0.001***	0.09
*nifH* probes (> 997 unique permutations, residual ECV = 12)				
Year	20.5 (1)	6.6	0.001***	0.6
Season	14.7 (1)	5.5	0.001***	0.4
Pond	21.1 (2)	7.8	0.001***	1.0
Location	4.3 (1)	2.7	0.006**	0.4
Time of day	4.0 (1)	2.6	0.007**	0.9
Depth	2.4 (1)	1.7	0.05*	0.6
Year × season	9.4 (1)	6.2	0.001***	0.3
*nosZ* probes (> 997 unique permutations, residual ECV = 16.9)				
Year	5.6 (1)	4.5	0.001***	0.2
Season	44.0 (1)	13.8	0.001***	0.001***
Pond	11.5 (2)	7.9	0.001***	0.05*
Location	5.9 (1)	4.7	0.001***	0.3
Time of day	3.8 (1)	3.5	0.006**	0.05*
Depth	1.1 (1)	0.7	0.4	0.8
Year × season	9.7 (1)	10.1	0.001***	0.001***

*df* degrees of freedom, *ECV* square root of estimates of components of variation indicating the effect as average % probe dissimilarity due to that factor. *P* value is based on >996 unique permutations; *PermDISP* permutational distance-based test for homogeneity of multivariate dispersions for main factors. ***P value = 0.001; **P value <0.01; *P value <0.05

### Relationships between N-Cycling Communities and the WSP Water Physico-Chemistry and Nutrients

**Fig. 4:**
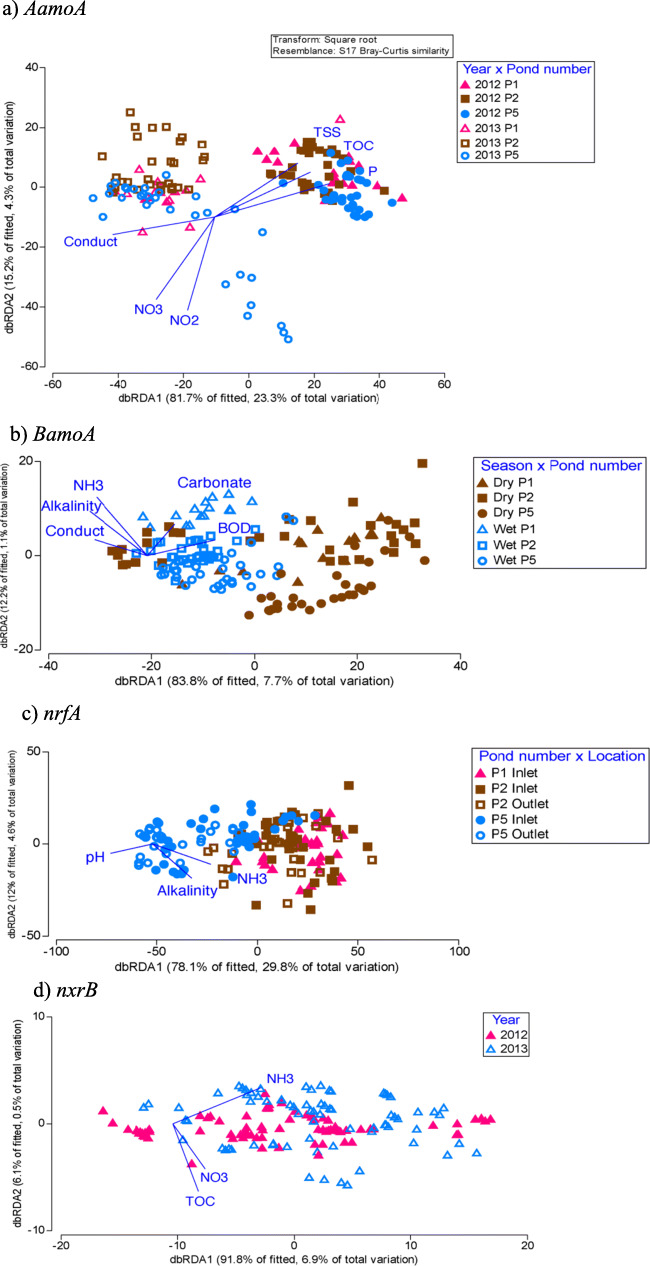
dbRDA plots of the nitrifying and DNRA communities and their relationship with N chemistry and physico-chemistry. Each nitrifying community is displayed according to the two most influential factors (year, season, pond, location, or time of day) as determined by PERMANOVA. The strength and direction of the relationship between abiotic factors and the community (or strictly speaking, the dbRDA axes) are shown with blue vectors. **a** *AamoA* community. **b** *BamoA* community. **c** *nrfA* community. **d** *nxrB* community. Dry, dry season; Wet, wet season; 2012, year 2012; 2013, year 2013; P1, pond 1; P2, pond 2; P5, pond 5

**Fig. 5:**
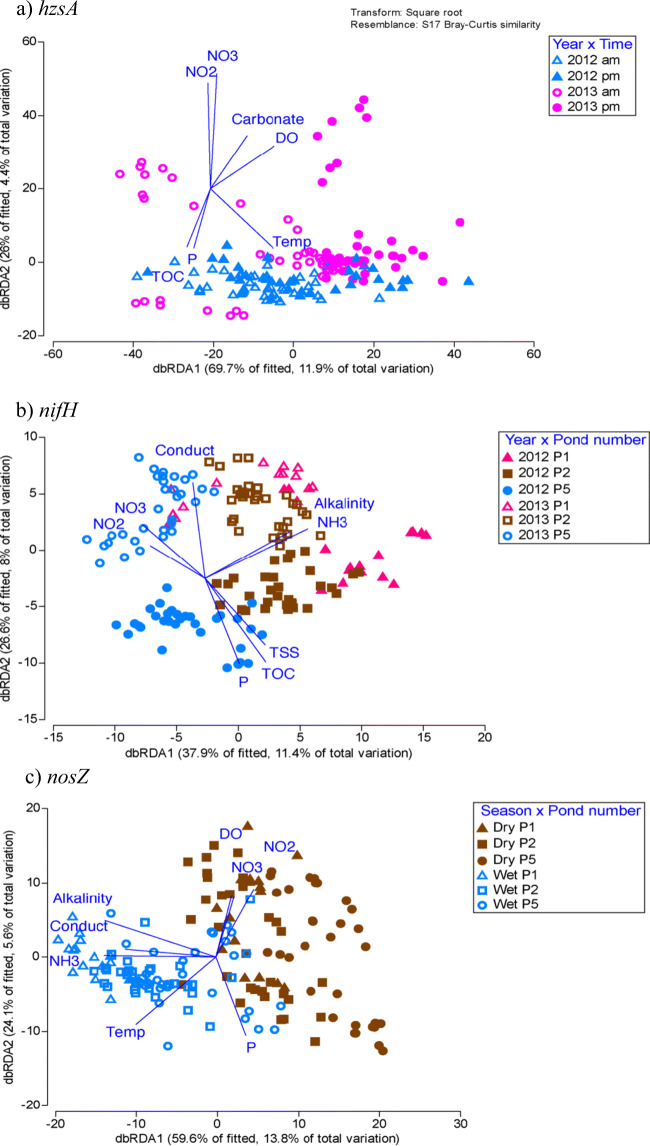
dbRDA plots of the denitrifying, anammox, and nitrogen fixation communities and their relationship with N chemistry and physico-chemistry. Each community is displayed according to the two most influential factors (year, season, pond, location, or time of day) as determined by PERMANOVA with 999 permutations. The strength and direction of the relationship between abiotic factors and the community (or strictly speaking, the dbRDA axes) are shown with blue vectors. **a** *hzsA* community. **b** *nifH* community. **c** *nosZ* community. Dry, dry season; Wet, wet season; 2012, year 2012; 2013, year 2013; P1, pond 1; P2, pond 2; P5, pond 5; am, morning; pm, afternoon

There were significant correlations between N-cycling communities and measured physico-chemistry and nutrients, and each functional community was correlated with different physico-chemical variables (Figs. 4 and 5). In general, the nitrifying and DNRA communities were correlated with wastewater environmental conditions, particularly alkalinity and ammonia (NH_3_) which were highest in ponds 1 and 2, especially during 2013 (Fig. 4 and Supplementary Tables 4 and 5). Conductivity and BOD levels were also correlated with *AamoA* and *BamoA* but were either weakly (*P* = 0.05) or not correlated to the *nrfA* and *nxrB* communities (Fig. 4 and Supplementary Table 4). For example, in 2012, *AamoA* communities were associated with high conductivity and low BOD concentrations while the opposite was true for 2013 (Fig. 4 and Supplementary Table 4). However, changes to the measured physico-chemistry explained <10% of the varying *BamoA* and *nxrB* and ~ 30% for the *AamoA* and *nrfA* communities (Fig. 4).

Similar to the nitrifying communities, the denitrifying, anammox, and nitrogen fixing communities were also strongly correlated to alkalinity and ammonia (NH_3_) (Figs. 4 and 5 and Supplementary Table 4). However, unlike the nitrifying community, the denitrifying and nitrogen fixing communities were correlated with concentrations of NO_2_^−^ and NO_3_^−^, which were higher in 2013 than in 2012 and higher in the dry season than in the wet season (Fig. 5 and Supplementary Tables 4 and 5).

### Yearly or Seasonal Pond Indicators for the Diverse Nitrogen Fixation and Denitrification Communities

**Fig. 6:**
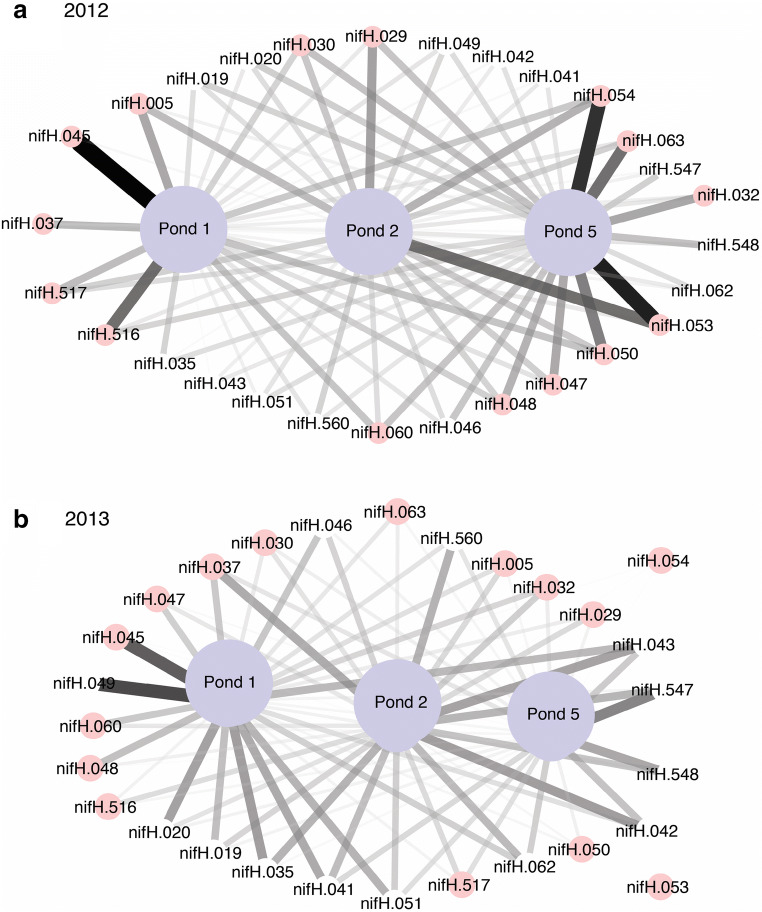
Cytoscape image for the 28 nitrogen fixation indicator probes for ponds in a 2012 and b 2013 as determined by IndVal. Each indicator probe was present in >90% of samples. Line thickness indicates the relative abundance of a positive probe in a pond, with thicker lines indicating a higher relative abundance in the pond. Indicators are grouped by the factors: pond number (ponds 1, 2, and 5) and year (2012, 2013) as chosen by the PERMANOVA analysis with 999 permutations. Pink circles, probes with high relative abundance for 2012

Unlike other functional groups, *nifH* and *nosZ* were represented by >40 different probes, and many of these probes were more prevalent in some ponds than others. IndVal was used to identify WSP pond indicator probes for 2012 and 2013. Of the 55 *nifH* probes detected in the WSP, 28 were present in 90% of all samples measured, and these were considered indicator candidates. *nifH* pond indicators were dynamic in that they significantly differed between ponds and years (Fig. 6). For example, with the exception of nifH.045, the *nifH* indicator probes that had a strong signal intensity in 2012 were weaker or absent in 2013 (Fig. 6). In addition, in 2012, pond 5 had a higher number (24) of indicators with strong signals than pond 1 (12), but in 2013, pond 1 had more (24) indicators than pond 5 (13) (Fig. 6).

**Fig. 7:**
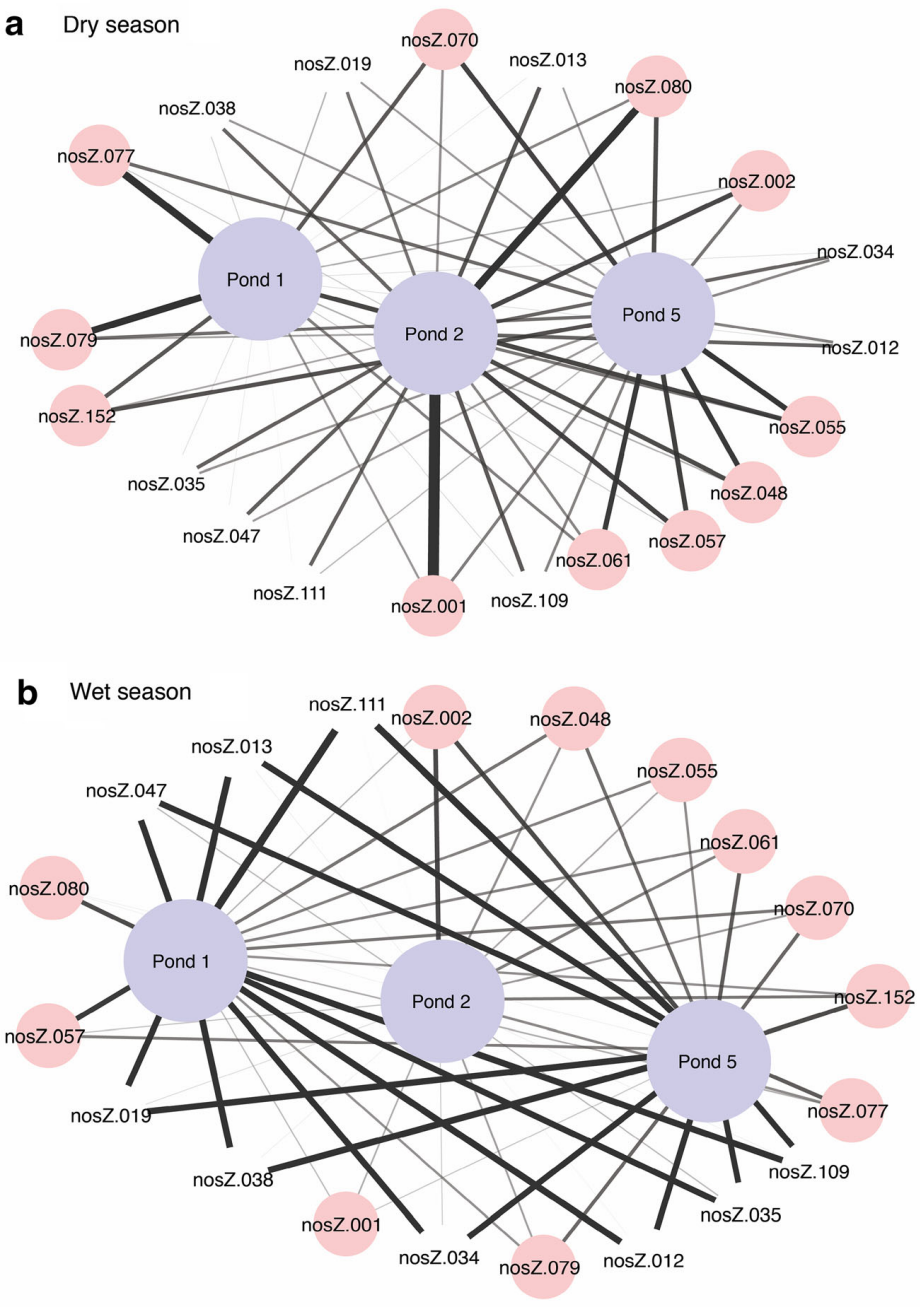
Cytoscape image for the 20 denitrification indicator probes for ponds during a the dry and b the wet seasons as determined by the IndVal analysis. Each indicator gene was present in >90% of samples. Line thickness indicates the relative abundance of a positive probe in a pond, with thicker lines indicating a higher relative abundance in the pond. Indicators are grouped by the factors: pond number (ponds 1, 2, and 5) and season (wet, dry) as chosen by the PERMANOVA analysis with 999 permutations. Pink circles, probes with high relative abundance for the dry season

Of the 47 *nosZ* probes present in the WSP, IndVal analysis identified 20 probes that were indicators for pond water (Fig. 7). As with *nifH* indicators, *nosZ* indicators also changed temporally. However, *nosZ* indicator genes changed seasonally rather than annually, with fewer indicator probes identified for ponds during the dry season than during the wet season (Fig. 7). Also, indicators that had a strong signal intensity during the dry season were not always positive for the wet season (Fig. 7). During the wet season, indicators for ponds 1 and 5 were similar (Fig. 7).

### WSP N-Cycling Gene Expression (cDNA)

With the exception of *nxrB*, gene expression signals were observed from the cDNA subset for all the N-cycling functional communities (Supplementary Fig. 2). For *BamoA* and *hzsA* communities, the same probes were positive for DNA and cDNA. For the other N-cycling communities, the number of probes positive for cDNA was less than the total number of positive DNA probes as follows (cDNA positive probes/total DNA probes): *nrfA* (4/5), *nifH* (41/55), *AamoA* three (3/6), and *nosZ* (21/47) (Supplementary Fig. 2). In general, *nosZ* and *nifH* probes with a strong positive signal for DNA were generally also positive for cDNA and were identified by the IndVal analysis as indicator candidates (Figs. 3, 6, and 7 and Supplementary Fig. 2). For example, positive *nifH* probes with strong signals like nifH – 019, 020, 051, and 062 hybridized for DNA and cDNA and were from the Gamma, Alpha, Beta, and Proteobacteria but not the Cyanobacteria (Supplementary Table 2 and Supplementary Fig. 2). Similarly, *nosZ* probes with strong signals for DNA, like nosZ – 070, 077, and 079, were also positive for cDNA (Fig. 3 and Supplementary Fig. 2). *nosZ* probes positive for >10 samples were from sediment clades (i.e. salt marsh, coastal sediment, activated sludge, and agricultural soil) and *Azospirillum* (Supplementary Table 2 and Supplementary Fig. 2).

## Discussion

As predicted, we found that the structure of each N-cycling community in the WSP shifted daily, seasonally, and yearly in response to changing wastewater conditions; however, the response of each community was not the same. The greatest changes to community composition were seen between years. Ammonia oxidizing bacterial genes (*BamoA*), that convert ammonia to nitrite, were the only exception to this yearly change, showing a strong presence in wet season samples only. Similar to our study, Short et al., (2013) also observed that *AamoA* and *BamoA* genes differed in community response to temporal change in an activated sludge plant. Interestingly, not all positive probes within a N-cycling community had the same general patterns. For example, in the dry season, different positive *nosZ* probes had opposite behaviours, where the number of positive nosZ – 043 (LS#1 - lake sediment clade #1) signals increased by 15%, while the number of nosZ – 057 (Agricult. soil clade #2) signals fell by 10%. In a study on a denitrification community, Babbin et al. [24] also found a complex and heterogeneous dynamic between individual genes and suggested that the heterogeneity was because of competition with other microbial communities. However, we found that competition between N-cycling groups may only explain a small part of the community change because N-cycling communities were only weakly correlated to each other. Instead, we propose that the heterogeneous response of individual probes is because of the different physiological responses bacteria and archaea evolve to cope with the environment and their interactions with other microbes [25]. Although not tested in this study, it is also possible that other microbes are competing with the N-cycling communities or that a bacterium that possesses a N-cycling gene may not necessarily utilize the gene, instead prioritizing the function of other genes. Our findings suggest that the N-cycling community patterns in the WSP are complex and change over time as communities interact with the environment and each other. Thus, characterizing a WSP based on a single snapshot in time would be misleading.

Contrary to our prediction, coupled FGA and nutrient chemistry data indicate that in a multi-pond system, different ponds harbour different N-cycling communities. While we expected there to be no difference in N-cycling population structure between the inlet and outlet of ponds, this was not the case for the measured communities, especially the nitrogen fixation (*nifH*), denitrification (*nosZ*), and dissimilatory nitrate reduction to ammonia (*nrfA*) communities. Instead, the diversity of these communities changed between ponds, as the waste progressed from ponds 1 to 5, with *nifH* and *nrfA* diversity increasing while *nosZ* diversity decreased. Dinitrogen is converted to ammonia by nitrogen fixation microbes. The highest *nifH* diversity, as shown by the highest average number of positive probes (45), was observed at the pond 1 inlet and coincided with the highest NH_3_ average (21.9 mg/L) measured. Again, the *nxrB* community that converts NO_2_^−^ to NO_3_^−^ was the only exception, with no significant differences in the number of positive probe signals between the ponds. Spatial change in N-cycling communities like *AamoA* has also been detected in other geographical-integrated surveys of wastewater treatment operations [15, 26]. Thus, because microbial communities are different in each pond, we recommend changing the current WSP influent/effluent monitoring regime to include all ponds.

We also predicted that nutrient concentrations could act as a surrogate for N-cycling community structure; however, this was not strongly supported. The anammox bacteria (*hzsA*) supported our prediction, where NH_3_, a known substrate utilized by the bacteria, was lowest in pond 5. The low ammonia concentration was associated with anammox bacteria, suggesting active consumption of the NH_3_ substrate. The influence of NH_3_ was also similar for the denitrifying *nosZ* community structure, which was also driven by the changing NH_3_ gradient rather than changes in NO_3_^−^ that the microbes utilize to convert NO_2_^−^, N_2_O, and finally N_2_ gas. These findings are contradictory to those of Fritz et al. [17] and Mayo and Abbas [3] who predicted that the rate of denitrification would be dependent on wastewater temperature and NO_3_^−^ concentration. Interestingly, we also found that *nifH* and *nrfA* bacterial groups could be predicted by the N chemistry they release. These two communities produce ammonia and had strong positive correlations to NH_3_ concentration. The highest numbers of positive *nifH* and *nrfA* probes were associated with pond 1, where ammonia was mainly concentrated. Instead of displaying a dependence on their known N substrate, the majority of N-cycling communities either positively or negatively correlated to the concentration of PO^4+^, which is another nutrient, many bacteria are speculated to depend upon [27]. The physico-chemistry also tended to influence the composition within a N-cycling community more than the N chemistry. For example, in the case of *hzsA*, DO was most influential to the community structure. There is increasing evidence that the relationship N-cycling microbes have with their N chemistry and physico-chemical environment is extremely complex, challenging previously accepted knowledge [28–30]. For example, a recent study on nitrifying bacteria showed that these bacteria may not be constrained to oxic conditions [28]. Thus, although N-cycling microbial community change was partially explained by changes to their environment, this relationship is complex and sometimes unpredictable. Given this complexity, measuring just the concentrations of N chemistry substrates and physico-chemistry is too simplistic and would hinder our ability to develop accurate knowledge of how WSP systems function. Therefore, it is likely direct measurements of N-cycling communities are needed to understand WSP efficiency.

The application of the FGA technology to include probes covering the entire nitrogen cycle enabled the simultaneous identification of the present N-cycling communities, as well as elucidating their expression. For example, FGA revealed that although *nxrB* DNA was present, this functional community was not active. Thus, since no *nxrB* activity was detected in the wastewater in this system, nitrite oxidation either was likely a chemical process (driven by wind action instead of bacteria) or was inhibited by active anammox bacteria [31–33]. However, we note that the lack of *nxrB* activity could be because the number of *nxrB* array probes is limited to the number of gene variants described in the literature or is a technical artefact created during the initial cDNA synthesis with random hexamers. Thus, to confirm if there is no *nxrB* expression requires further investigation with more samples. Additionally, research indicates that the presence of a N-cycling gene does not mean the bacterium is limited to N chemistry for survival. The ability for bacteria to survive on multiple substrates could also explain why the 2nd-stage analysis of the N-cycling communities in the ponds indicated patterns of N-cycling groups were not dependent on each other, despite literature predicting otherwise [26, 34–36]. Thus, FGA technology is both an exploratory and a practical tool for WSPs and also has strong applications to a wide array of ecosystems for N-cycling identification in future.

The WSP has a unique N-cycling fingerprint, which is dynamic over time and space, and this has implications for management. Because of the complex patterns of N-cycling functional communities, it would be valuable to perform microcosm experiments, targeting genes which were both expressed and responded to changes in the physico-chemistry and N nutrients, to further quantify and explore their relationships. Short et al. [9] also found merit in applying broad-spectrum ecological tools, like the FGA, to identify important bacterial communities of interest in an activated sludge system. The study found that environmental niche preferences could favour some functional groups over others and thus affect the community ecology and diversity. Thus, it is important to consider all microbial and chemical aspects that impact a WSP, so that critical information is not missed when characterizing and understanding functional ecology and pond processes. Future application of the FGA will allow managers to monitor the N-cycling health of the WSP and improved general understanding to make appropriate decisions to enhance N-removal efficiency.

## Conclusion

N-cycling functional communities showed a complex relationship with the yearly, seasonal, and daily timing and location of sampling, as indicated by the lack of general trends between the communities. Identifying clear community patterns was further complicated by the fact that genes within a community also displayed individual and often opposite responses over time and between ponds. Because microbial communities were different in each pond, we recommend changing the current WSP influent/effluent sampling regime to include all ponds. The weak relationships identified between different N-cycling communities were likely partially because of the affinity microbes had to wastewater physico-chemistry and N chemistry. However, the changing chemistry alone could not adequately explain community patterns in the WSP. Only the anammox bacteria (*hzsA*) supported our hypothesis that N chemistry could act as a surrogate for N-cycling communities. These data indicate the necessity of taking direct DNA and cDNA measurements of N microbes to understand WSP efficiency. These data also provided insight about why it is difficult to manage these microbes through large-scale manipulation of the wastewater environment, as their community composition is dependent on multiple factors and conditions. Overall, we found FGA technology a useful exploratory and practical tool for WSPs with strong applications to a wide array of ecosystems for N-cycling identification in future. In addition, the FGA can be used for monitoring the N-cycling health of a WSP and for developing an N budget, which would lead to informed management decisions that enhance N removal efficiency.

## Supplementary Information

ESM 1 (DOCX 22626 kb)

ESM 2 (XLSX 106 kb)

ESM 3 (XLSX 29 kb)

## Data Availability

Available as supplementary material.
