# Supplementary material for: The Diversity of Nitrogen-Cycling Microbial Genes in a Waste Stabilization Pond Reveals Changes over Space and Time that Is Uncoupled to Changing Nitrogen Chemistry
Source: Microb Ecol. 2020 Nov 10;81(4):1029–41. doi: 10.1007/s00248-020-01639-x (PMC8062326; doi:10.1007/s00248-020-01639-x)
Supplement: Supplementary file 1 — (DOCX 22626 kb) [file 248_2020_1639_MOESM1_ESM.docx]

**Supplementary material**

**Methods**

**Supplementary Table 1** PCR primers and fragment lengths for the N-cycle array.

| Target | Primer | Sequence | Fragment size (bp) | Reference |
| --- | --- | --- | --- | --- |
| A-*amoA* | Arch amoA-1F | STAATGGTCTGGCTTAGACG | 635 | Francis et al. (2005) |
|  | Arch amoA-2R-T7 | TAATACGACTCACTATAGGCGGCCATCCATCTGTATGT |  |  |
| B-*amoA* | amoA-1F | GGGGTTTCTACTGGTGGT | 490 | Rotthauwe et al. (1997) |
|  | amoA-2R-T7 | TAATACGACTCACTATAGCCCCTCKGSAAAGCCTTCTTC |  |  |
| *hzsA* primary fragment | hzsA_382F | GGYGGDTGYCAGATATGGG | 2000 | Harhangi et al. (2012) |
|  | hzsA_2390R | ATRTTRTCCCAYTGYGCHCC |  |  |
| *hzsA* final fragment | hzsA_526F | TAYTTTGAAGGDGACTGG | 1331 | Harhangi et al. (2012) |
|  | hzsA_1857R-T7 | TAATACGACTCACTATAGAAABGGYGAATCATARTGGC |  |  |
| *nifH* | nifH2_F | GYGAYCCNAARGCNGA | 362 | Zehr and McReynolds (1989) |
|  | nifH1_R-T7 | TAATACGACTCACTATAGADNGCCATCATYTCNCC |  |  |
| *nosZ* | nosZ-F | CGYTGTTCMTCGACAGCCAG | 453 | Kloos et al. (2001) |
|  | nosZ 1622R-T7 | TAATACGACTCACTATAGCGSACCTTSTTGCCSTYGCG |  | Throbäck et al. (2004) |
| *nrfA* | nrfA_F1 | GCNTGYTGGWSNTGYAA | 490 | Mohan et al. (2004) |
|  | nrfA_R1-T7 | TAATACGACTCACTATAGTWNGGCATRTGRCARTC |  |  |
| *nxrB* -  Nitrobacter | nxrB1F | ACGTGGAGACCAAGCCGGG | 380 | Vanparys et al. (2007) |
|  | nxrB1R-T7 | TAATACGACTCACTATAGCCGTGCTGTTGAYCTCGTTGA |  |  |
| T7 RNA polymerase promoter tag | T7 | TAATACGACTCACTATAG |  |  |

**Results**

**WSP N-cycling gene diversity (DNA)**

FGA measurements of the ammonia oxidising archaeal DNA identified that six of the 60 tested *AamoA* probes were positive (Figure 3). The *AamoA* probes belong to the Archaea: *C. symbiosum et rel* (AamoA-32); *N. spharea* or *N. caldus* (*AamoA* – 87, 85, & 86); and *Soil fosmid 54d9 lineage* (*AamoA* – 54 & 55; Figure 3 & Supplementary Table 2). Of the 99 *BamoA* probes tested, a total of seven were identified as ammonia oxidising bacteria from the taxa *Nitrosomonas* (*BamoA – 29* & *31*) and *Broad Uncultured AOBI* (*BamoA – 12, 07, 10, 08* & *09*; Figure 3 & Supplementary Table 2). For *nxrB,* a total of eight (21) *nxrB* probes were identified in the DNA, of which were all *Nitrobacter* except *nxrB – 018*, which was a LB5a clade (Figure 3 & Supplementary Table 2). The five positive *nrfA* probes were; *nrfA – 01, 02, 03, 21 & 98*, are associated with *Escherichia coli*, *Aeromonas* (*nrfA – 21*) and CT clade sequences from coal tar waste (*nrfA – 98*) (Supplementary Table 2). Six of the positive Anammox community probes were from *Brocardia fulgida* subgroups, while the *hzsA – 004* probe was from *Brocardia anammoxidans* and the n-DAMO clade, and *hzsA – 006* from another n-DAMO clade (Supplementary Table 2). The nitrogen fixing community (*nifH*) were represented by 55 (of 144 tested) probes (Figure 3). Of the 55 signals, 56.4% were Gammaproteobacteria, 21.8% Alpha- and Betaproteobacteria and 21.8% were Cyanobacteria (Supplementary Table 2). The positive *nosZ* probes were represented by many different taxa, with *Rhizobiales* the most common taxa, representing 13 out of the 47 positive probes (Supplementary Table 2).

**Supplementary Table 2.** Positive N-cycle probes and their associated taxa identification.

| **Probe** | **Species ID** |
| --- | --- |
| BamoA 29 | *Nitrosomonas* |
| BamoA 31 | *Nitrosomonas* |
| BamoA 12 | Broad Unc AOBI |
| BamoA 07 | Broad Unc AOBI |
| BamoA 10 | Broad Unc AOBI |
| BamoA 08 | Broad Unc AOBI |
| BamoA 09 | Broad Unc AOBI |
| AamoA 32 | *C. symbiosum et rel*, Dsed2 |
| AamoA 87 | *N. sphaera / N. caldus* |
| AamoA 85 | *N. sphaera / N. caldus* |
| AamoA 86 | *N. sphaera / N. caldus* |
| AamoA 54 | Soil fosmid 54d9 lineage |
| AamoA 55 | Soil fosmid 54d9 lineage |
| nxrB 001 | *Nitrobacter* |
| nxrB 002 | *Nitrobacter* |
| nxrB 003 | *Nitrobacter* |
| nxrB 007 | *Nitrobacter* |
| nxrB 004 | *Nitrobacter* |
| nxrB 006 | *Nitrobacter* |
| nxrB 005 | *Nitrobacter* |
| nxrB 018 | LB5a clade |
| nrfA 01 | *E. coli* |
| nrfA 02 | *E. coli* |
| nrfA 03 | *E. coli* |
| nrfA 21 | *Aeromonas* |
| nrfA 98 | CT clade (sequences from coal tar waste) |
| nosZ 012 | *Rhizobiales* |
| nosZ 013 | *Rhizobiales* |
| nosZ 019 | *Rhizobiales* |
| nosZ 020 | *Rhizobiales* |
| nosZ 024 | *Rhizobiales* |
| nosZ 025 | *Rhizobiales* |
| nosZ 038 | *Rhizobiales* |
| nosZ 033 | *Rhizobiales* |
| nosZ 035 | *Rhizobiales* |
| nosZ 031 | *Rhizobiales* |
| nosZ 034 | *Rhizobiales* |
| nosZ 111 | *Rhizobiales* |
| nosZ 109 | *Rhizobiales* |
| nosZ 016 | *Brucella* |
| nosZ 018 | *Brucella* |
| nosZ 043 | LS#1 - Lake sediment clade #1 |
| nosZ 044 | LS#1 - Lake sediment clade #1 |
| nosZ 045 | FS#1 - Forest soil clade #1 |
| nosZ 046 | FS#1 - Forest soil clade #1 |
| nosZ 047 | FS#1 - Forest soil clade #1 |
| nosZ 001 | *Azospirillum* + Salt Marsh clade #1 |
| nosZ 002 | *Azospirillum* + Salt Marsh clade #1 |
| nosZ 048 | Salt Marsh clade #1 |
| nosZ 049 | *Nisea* related |
| nosZ 050 | *Nisea* related |
| nosZ 159 | Coastal sediment clade #1 |
| nosZ 157 | *M. cystis* related |
| nosZ 145 | UPs1 clade |
| nosZ 056 | *Herbaspirillum* |
| nosZ 058 | Agricult. soil clade #2 |
| nosZ 057 | Agricult. soil clade #2 |
| nosZ 055 | *Herbaspirillum* |
| nosZ 061 | Paddy Soil #1 |
| nosZ 069 | UPs2 |
| nosZ 070 | *Azospirillum largimobile* |
| nosZ 067 | *Aromatoleum* |
| nosZ 077 | Agricult. soil clade #4 |
| nosZ 079 | Activated sludge |
| nosZ 080 | Lake sediment #3 |
| nosZ 089 | UPs3 |
| nosZ 091 | UPs3 |
| nosZ 152 | Activated sludge |
| nosZ 139 | *Sinorhizobium* and rel. |
| nosZ 163 | Unknown |
| nosZ 123 | *Ruegeria* |
| nosZ 121 | *Silicibacter* |
| nosZ 064 | *Burkholderia* |
| hzsA 010 | *Brocardia fulgida* subgroup 2 |
| hzsA 011 | *Brocardia fulgida* subgroup 2 |
| hzsA 015 | *Brocardia fulgida* subgroup 4 + 7 + 9 |
| hzsA 016 | *Brocardia fulgida* subgroup 4 + 7 + 9 |
| hzsA 038 | *Brocardia fulgida* subgroup 4 + 7 |
| hzsA 042 | *Brocardia fulgida* subgroup 9 |
| hzsA 004 | *Brocardia* *anammoxidans* + n-DAMO clade |
| hzsA 006 | n-DAMO clade |
| nifH 007 | Cyanobacteria -1B |
| nifH 005 | *Cyanobacteria -1B Synechococcus + Acaryochloris* |
| nifH 004 | *Cyanobacteria -1B Synechococcus + Acaryochloris* |
| nifH 006 | Cyanobacteria -1B |
| nifH 560 | *Cyanobacteria -1B Synechococcus + Acaryochloris* |
| nifH 547 | Cyanobacteria -1B |
| nifH 548 | Cyanobacteria -1B |
| nifH 055 | Cyanobacteria -1B |
| nifH 516 | γ Proteobacteria, 1G, *Azotobacter* |
| nifH 573 | Cyanobacteria -1B |
| nifH 574 | Cyanobacteria -1B |
| nifH 565 | Cyanobacteria -1B |
| nifH 522 | Cyanobacteria -1B |
| nifH 041 | γ Proteobacteria, 1G, *Azotobacter* |
| nifH 043 | γ Proteobacteria, 1G, *Azotobacter* |
| nifH 042 | γ Proteobacteria, 1G, *Azotobacter* |
| nifH 050 | α + β Proteobact- 1J |
| nifH 051 | α + β Proteobact- 1J- Marine surface clade |
| nifH 052 | α + β Proteobact- 1J- Marine surface clade |
| nifH 053 | α + β Proteobact- 1J- Marine surface clade |
| nifH 054 | α + β Proteobact- 1J- Marine surface clade |
| nifH 046 | α + β Proteobact- 1J |
| nifH 047 | α + β Proteobact- 1K |
| nifH 048 | α + β Proteobact- 1K |
| nifH 049 | α + β Proteobact- 1K |
| nifH 032 | α + β Proteobact- 1K, Marine microbial mat clade, Intertidal microbial mat |
| nifH 062 | γ Proteobacteria, 1P, S.Pacific gyre |
| nifH 517 | α + β Proteobact- 1K |
| nifH 060 | γ Proteobacteria, 10, Mangrove rhizosphere clade |
| nifH 501 | α + β Proteobact- 1K |
| nifH 037 | γ Proteobacteria, 1A |
| nifH 057 | γ Proteobacteria, 10, SPOT plankton clade |
| nifH 515 | γ Proteobacteria, 10 |
| nifH 044 | γ Proteobacteria, 10 |
| nifH 045 | γ Proteobacteria, 10, *Thiorodovibrio* |
| nifH 039 | γ Proteobacteria, 1G, *Azotobacter* |
| nifH 040 | γ Proteobacteria, 1G |
| nifH 520 | γ Proteobacteria, 1G |
| nifH 519 | γ Proteobacteria, 1G |
| nifH 020 | γ Proteobacteria, 1P |
| nifH 019 | γ Proteobacteria, 1P |
| nifH 568 | γ Proteobacteria, 1G |
| nifH 507 | γ Proteobacteria, 1G |
| nifH 061 | γ Proteobacteria, 1G |
| nifH 506 | γ Proteobacteria, 1P, *Dechloromonas aromatica,* Chesapeake Bay phylotype |
| nifH 036 | γ Proteobacteria, 1P |
| nifH 035 | γ Proteobacteria, 1A |
| nifH 064 | γ Proteobacteria, 1A |
| nifH 065 | γ Proteobacteria, 1A |
| nifH 518 | γ Proteobacteria, 1A |
| nifH 063 | γ Proteobacteria, 1A |
| nifH 030 | γ Proteobacteria, 1A |
| nifH 029 | γ Proteobacteria, 1A |
| nifH 023 | γ Proteobacteria, 1C |
| nifH 510 | γ Proteobacteria, 1C |

**Supplementary Table 3.** Spearman’s ranked 2^nd^ Stage analysis of the seven functional N-cycling communities. Correlation = -1 to 1.

|  | *nxrB* | *AamoA* | *BamoA* | *hzsA* | *nifH* | *nosZ* | *nrfA* |
| --- | --- | --- | --- | --- | --- | --- | --- |
| *nxrB* |  |  |  |  |  |  |  |
| *AamoA* | -0.031 |  |  |  |  |  |  |
| *BamoA* | -0.035 | 0.011 |  |  |  |  |  |
| *hzsA* | 0.008 | -0.001 | 0.041 |  |  |  |  |
| *nifH* | -0.054 | 0.098 | 0.071 | 0.100 |  |  |  |
| *nosZ* | -0.040 | 0.031 | 0.043 | 0.151 | 0.382 |  |  |
| *nrfA* | 0.028 | -0.003 | 0.016 | 0.043 | 0.233 | 0.124 |  |

A)

B)

C)

**Supplementary Figure 1.** Heatmaps of *BamoA, AamoA, nxrB*, *nrfA*, *nosZ, hzsA* and *nifH* FGA DNA in Ponds 1, 2 and 5. A value of 100 means the signal was equal to that of the control probe (hyaBP60), whereas a value of 10 indicates that the signal was 10 % of the control. Colour coding is indicated on the colour bar on top of heatmap. See Supplementary Table 2 for probe label and taxa identification details. N-cycling communities in each pond arranged by year (2012 and 2013) (A), season (dry and wet) (B) and time of day (morning and afternoon) (C). P1 = pond 1, P2 = pond 2, P5 = pond 5.

**Relationships between N-cycling functional groups and water physico-chemistry and nutrients**

**Supplementary Table 4.** DistLM marginal tests for N-cycling bacterial communities with the measured physico-chemistry and nutrients. Similarity matrix = Bray Curtis, residual df = 158.

| ***AamoA*** | | | | |
| --- | --- | --- | --- | --- |
| Variable | SS (trace) | Pseudo-F | P | Prop. |
| DO | 4395.9 | 1.3 | 0.259 | 0.008 |
| Conduct | 70983.0 | 24.4 | 0.001 | 0.134 |
| Temp | 10683.0 | 3.2 | 0.035 | 0.020 |
| pH | 8879.0 | 2.7 | 0.051 | 0.017 |
| TSS | 51457.0 | 17.0 | 0.001 | 0.097 |
| BOD | 35430.0 | 11.3 | 0.001 | 0.067 |
| TOC | 72776.0 | 25.1 | 0.001 | 0.137 |
| Alkalinity | 13145.0 | 4.0 | 0.020 | 0.025 |
| Carbonate | 13715.0 | 4.2 | 0.011 | 0.026 |
| NH3 | 12468.0 | 3.8 | 0.022 | 0.023 |
| NO2 | 17837.0 | 5.5 | 0.003 | 0.034 |
| NO3 | 36401.0 | 11.6 | 0.001 | 0.069 |
| N | 2923.7 | 0.9 | 0.498 | 0.006 |
| P | 89726.0 | 32.2 | 0.001 | 0.169 |
| ***BamoA*** | | | | |
| Variable | SS (trace) | Pseudo-F | P | Prop. |
| DO | 2755.9 | 0.84923 | 0.464 | 0.005 |
| Conduct | 11063 | 3.4651 | 0.007 | 0.021 |
| Temp | 6605 | 2.0507 | 0.089 | 0.013 |
| pH | 1181.2 | 0.36286 | 0.841 | 0.002 |
| TSS | 3534.8 | 1.0909 | 0.355 | 0.007 |
| BOD | 25442 | 8.2027 | 0.001 | 0.049 |
| TOC | 1074.9 | 0.33016 | 0.882 | 0.002 |
| Alkalinity | 22823 | 7.3192 | 0.001 | 0.044 |
| Carbonate | 6355.9 | 1.9724 | 0.092 | 0.012 |
| NH3 | 17333 | 5.4973 | 0.002 | 0.034 |
| NO2 | 6296.5 | 1.9537 | 0.101 | 0.012 |
| NO3 | 3164.6 | 0.97594 | 0.417 | 0.006 |
| N | 6100.5 | 1.8922 | 0.081 | 0.012 |
| P | 9778.5 | 3.0551 | 0.025 | 0.019 |
| ***nrfA*** | | | | |
| Variable | SS (trace) | Pseudo-F | P | Prop. |
| DO | 2327 | 0.77605 | 0.448 | 0.005 |
| Conduct | 7330 | 2.4706 | 0.083 | 0.015 |
| Temp | 2371.9 | 0.79108 | 0.463 | 0.005 |
| pH | 36271 | 13.03 | 0.001 | 0.076 |
| TSS | 6479.4 | 2.1799 | 0.089 | 0.014 |
| BOD | 8696.4 | 2.9397 | 0.053 | 0.018 |
| TOC | 5938.2 | 1.9956 | 0.130 | 0.012 |
| Alkalinity | 36029 | 12.935 | 0.001 | 0.076 |
| Carbonate | 1134.5 | 0.37741 | 0.745 | 0.002 |
| NH3 | 70845 | 27.621 | 0.001 | 0.149 |
| NO2 | 2779 | 0.92766 | 0.411 | 0.006 |
| NO3 | 2887.7 | 0.96418 | 0.356 | 0.006 |
| N | 22737 | 7.9241 | 0.001 | 0.048 |
| P | 9830.1 | 3.331 | 0.034 | 0.021 |
| ***nxrB*** | | | | |
| Variable | SS (trace) | Pseudo-F | P | Prop. |
| DO | 267.82 | 0.32821 | 0.694 | 0.002 |
| Conduct | 2839.1 | 3.5502 | 0.035 | 0.022 |
| Temp | 578.62 | 0.71082 | 0.449 | 0.004 |
| pH | 253.68 | 0.31085 | 0.702 | 0.002 |
| TSS | 292.19 | 0.35816 | 0.684 | 0.002 |
| BOD | 984.3 | 1.213 | 0.276 | 0.008 |
| TOC | 597.61 | 0.73426 | 0.415 | 0.005 |
| Alkalinity | 4607 | 5.8427 | 0.009 | 0.036 |
| Carbonate | 58.405 | 0.07146 | 0.962 | 0.000 |
| NH3 | 6571.3 | 8.4673 | 0.004 | 0.051 |
| NO2 | 259.39 | 0.31787 | 0.688 | 0.002 |
| NO3 | 310.38 | 0.38051 | 0.654 | 0.002 |
| N | 238.32 | 0.292 | 0.416 | 0.002 |
| P | 1259.4 | 1.5554 | 0.213 | 0.010 |
| ***hzsA*** | | | | |
| Variable | SS (trace) | Pseudo-F | P | Prop. |
| DO | 20204 | 7.5262 | 0.002 | 0.045 |
| Conduct | 8254.2 | 2.9905 | 0.047 | 0.019 |
| Temp | 13618 | 4.9955 | 0.007 | 0.031 |
| pH | 11828 | 4.3209 | 0.013 | 0.027 |
| TSS | 4608.6 | 1.6559 | 0.185 | 0.010 |
| BOD | 165.05 | 0.058711 | 0.983 | 0.000 |
| TOC | 8658.7 | 3.14 | 0.040 | 0.019 |
| Alkalinity | 10808 | 3.9387 | 0.019 | 0.024 |
| Carbonate | 7587.5 | 2.7448 | 0.041 | 0.017 |
| NH3 | 12837 | 4.7004 | 0.008 | 0.029 |
| NO2 | 15600 | 5.7488 | 0.008 | 0.035 |
| NO3 | 17841 | 6.6091 | 0.004 | 0.040 |
| N | 186.47 | 0.066332 | 0.994 | 0.000 |
| P | 5145.7 | 1.8511 | 0.155 | 0.012 |
| ***nifH*** | | | | |
| Variable | SS (trace) | Pseudo-F | P | Prop. |
| DO | 1217 | 4.0113 | 0.003 | 0.025 |
| Conduct | 3295.6 | 11.355 | 0.001 | 0.067 |
| Temp | 1929.8 | 6.4567 | 0.001 | 0.039 |
| pH | 2003.2 | 6.7128 | 0.001 | 0.041 |
| TSS | 2690.6 | 9.1497 | 0.001 | 0.055 |
| BOD | 1753.3 | 5.8444 | 0.001 | 0.036 |
| TOC | 3387.5 | 11.695 | 0.001 | 0.069 |
| Alkalinity | 3578.7 | 12.407 | 0.001 | 0.073 |
| Carbonate | 1426.8 | 4.7236 | 0.001 | 0.029 |
| NH3 | 4545.2 | 16.099 | 0.001 | 0.092 |
| NO2 | 2206.9 | 7.4274 | 0.001 | 0.045 |
| NO3 | 3161.4 | 10.861 | 0.001 | 0.064 |
| N | 1131.1 | 3.7217 | 0.029 | 0.023 |
| P | 3067.3 | 10.516 | 0.001 | 0.062 |
| ***nosZ*** | | | | |
| Variable | SS (trace) | Pseudo-F | P | Prop. |
| DO | 2588.7 | 3.4543 | 0.013 | 0.021 |
| Conduct | 8510.2 | 11.954 | 0.001 | 0.070 |
| Temp | 8240.1 | 11.546 | 0.001 | 0.068 |
| pH | 2192.2 | 2.9155 | 0.022 | 0.018 |
| TSS | 3795.4 | 5.1166 | 0.002 | 0.031 |
| BOD | 3841.5 | 5.1808 | 0.002 | 0.032 |
| TOC | 2047.9 | 2.7203 | 0.024 | 0.017 |
| Alkalinity | 12086 | 17.533 | 0.001 | 0.100 |
| Carbonate | 2204.7 | 2.9324 | 0.022 | 0.018 |
| NH3 | 11907 | 17.245 | 0.001 | 0.098 |
| NO2 | 3213.3 | 4.3105 | 0.009 | 0.027 |
| NO3 | 2735 | 3.654 | 0.015 | 0.023 |
| N | 800.72 | 1.0526 | 0.191 | 0.007 |
| P | 5138 | 7.0069 | 0.001 | 0.042 |

**Supplementary Table 5.** Average (Av) measured physico-chemistry and N-chemistry values ± standard deviation (SD). Dry = dry season; Wet = wet season; 2012= year 2012; 2013 = year 2013; P1 = Pond 1; P2 = Pond 2; P5 = Pond 5.

| Factor | | | Variable | | | | | | | | | | | | | | | | | | | | | | | | |
| --- | --- | --- | --- | --- | --- | --- | --- | --- | --- | --- | --- | --- | --- | --- | --- | --- | --- | --- | --- | --- | --- | --- | --- | --- | --- | --- | --- |
|  |  |  | DO mg/L | Conductivity (S/m) | Temperature ^o^C | | pH | | TSS mg/L | | BOD mg/L | | TOC mg/L | | Alkalinity mg/L | | Carbonate mg/L | | NH_3_ mg/L | | NO_2_^-^ mg/L | | NO_3_^-^ mg/L | | N mg/L | | P mg/L |
| Year | 2012 | Av ± SD | 4.6 ± 5.1 | 540.2 ± 42.6 | 31.6 ± 2.7 | 7.8 ± 0.8 | | 204.8 ± 32.3 | | 122.4 ± 40.6 | | 92.0 ± 11.9 | | 130.1 ± 24.9 | | 0.2 ± 0.1 | | 17.1 ± 6.01 | | 0.006 ± 0.003 | | 0.008 ± 0.006 | | 34.08 ± 5.003 | | 6.9 ± 0.6 | |
|  | 2013 | Av ± SD | 5.6 ± 5.9 | 669.3 ± 79.7 | 28.0 ± 4.6 | 7.8 ± 0.6 | | 147.9 ± 30.8 | | 73.9 ± 19.8 | | 59.01 ± 19.5 | | 140.8 ± 20.4 | | 0.3 ± 0.2 | | 18.3 ± 6.1 | | 0.6 ± 0.8 | | 0.3 ± 0.2 | | 36.4 ± 17.1 | | 4.0 ± 0.6 | |
| Season | Dry | Av ± SD | 6.7 ± 6.3 | 551.6 ± 53.5 | 27.0 ± 3.8 | 7.8 ± 0.8 | | 194.3 ± 41.0 | | 114.2 ± 48.5 | | 79.9 ± 20.6 | | 121.6 ± 21.4 | | 0.3 ± 0.2 | | 14.6 ± 5.8 | | 0.5 ± 0.8 | | 0.2 ± 0.3 | | 36.5 ± 16.8 | | 5.5 ± 2.0 | |
|  | Wet | Av ± SD | 3.4 ± 3.9 | 657.9 ± 89.5 | 32.6 ± 2.2 | 7.8 ± 0.6 | | 158.4 ± 36.0 | | 82.2 ± 18.8 | | 71.2 ± 24.8 | | 149.3 ± 15.7 | | 0.2 ± 0.1 | | 20.8 ± 4.6 | | 0.1 ± 0.1 | | 0.1 ± 0.1 | | 34.0 ± 5.7 | | 5.4 ± 1.0 | |
| Pond | P1 | Av ± SD | 5.0 ± 5.7 | 624.1 ± 84.7 | 30.1 ± 3.6 | 7.4 ± 0.6 | | 182.3 ± 47.7 | | 113.0 ± 42.0 | | 73.3 ± 23.5 | | 151.9 ± 20.5 | | 0.3 ± 0.2 | | 21.9 ± 6.7 | | 0.3 ± 0.5 | | 0.1 ± 0.1 | | 41.6 ± 24.2 | | 5.5 ± 1.6 | |
|  | P2 | Av ± SD | 4.4 ± 4.7 | 600.7 ± 88.3 | 30.1 ± 4.0 | 7.6 ± 0.6 | | 187.6 ± 41.3 | | 98.7 ± 47.6 | | 82.7 ± 21.8 | | 141.3 ± 17.0 | | 0.2 ± 0.1 | | 20.7 ± 2.8 | | 0.1 ± 0.1 | | 0.1 ± 0.1 | | 37.6 ± 5.6 | | 5.4 ± 1.5 | |
|  | P5 | Av ± SD | 5.8 ± 6.1 | 599.1 ± 96.2 | 29.3 ± 4.6 | 8.1 ± 0.7 | | 162.2 ± 37.1 | | 90.2 ± 27.2 | | 69.5 ± 22.6 | | 121.4 ± 22.4 | | 0.2 ± 0.2 | | 12.6 ± 4.3 | | 0.5 ± 0.9 | | 0.2 ± 0.3 | | 29.7 ± 4.7 | | 5.5 ± 1.7 | |
| Time-of-day | 6am | Av ± SD | 1.8 ± 2.0 | 608.2 ± 88.8 | 28.3 ± 4.2 | 7.4 ± 0.4 | | 175.1 ± 43.3 | | 90.7 ± 38.4 | | 77.2 ± 20.9 | | 138.3 ± 24.5 | | 0.2 ± 0.1 | | 18.0 ± 6.3 | | 0.3 ± 0.5 | | 0.1 ± 0.2 | | 36.5 ± 16.4 | | 5.5 ± 1.6 | |
|  | 1pm | Av ± SD | 8.4 ± 5.9 | 601.3 ± 93.3 | 31.3 ± 3.7 | 8.1 ± 0.8 | | 177.6 ± 41.9 | | 105.7 ± 40.5 | | 73.9 ± 25.1 | | 132.6 ± 21.8 | | 0.3 ± 0.2 | | 17.4 ± 5.9 | | 0.4 ± 0.7 | | 0.2 ± 0.2 | | 34.0 ± 6.7 | | 5.4 ± 1.6 | |
| Location | Inlet | Av ± SD | 5.8 ± 5.6 | 606.0 ± 90.1 | 30.2 ± 4.4 | 7.7 ± 0.8 | | 182.0 ± 43.3 | | 102.5 ± 42.1 | | 77.0 ± 23.4 | | 140.7 ± 23.4 | | 0.2 ± 0.2 | | 19.1 ± 6.2 | | 0.3 ± 0.7 | | 0.1 ± 0.2 | | 36.9 ± 15.0 | | 5.5 ± 1.6 | |
|  | Outlet | Av ± SD | 4.1 ± 5.2 | 602.8 ± 92.6 | 29.2 ± 3.9 | 7.8 ± 0.6 | | 167.8 ± 40.0 | | 91.7 ± 36.1 | | 73.3 ± 22.7 | | 127.6 ± 21.0 | | 0.2 ± 0.2 | | 15.6 ± 5.2 | | 0.3 ± 0.5 | | 0.2 ± 0.2 | | 32.7 ± 7.0 | | 5.5 ± 1.6 | |
| Pond/time | P1 6am | Av ± SD | 3.6 ± 3.2 | 623.0 ± 87.1 | 29.3 ± 4.0 | 7.2 ± 0.4 | | 180.2 ± 50.9 | | 104.9 ± 42.9 | | 74.0 ± 25.7 | | 158.1 ± 17.5 | | 0.2 ± 0.1 | | 22.9 ± 6.9 | | 0.4 ± 0.7 | | 0.2 ± 0.2 | | 47.8 ± 33.2 | | 5.5 ± 1.9 | |
|  | P1 1pm | Av ± SD | 6.3 ± 7.3 | 625.2 ± 84.9 | 31.0 ± 3.0 | 7.7 ± 0.8 | | 184.4 ± 45.8 | | 121.1 ± 40.8 | | 72.7 ± 21.9 | | 145.7 ± 21.9 | | 0.3 ± 0.2 | | 20.9 ± 6.7 | | 0.2 ± 0.2 | | 0.1 ± 0.1 | | 35.4 ± 5.6 | | 5.5 ± 1.4 | |
|  | P2 6am | Av ± SD | 1.4 ± 1.2 | 605.5 ± 82.7 | 28.4 ± 4.0 | 7.4 ± 0.4 | | 183.5 ± 40.9 | | 86.9 ± 43.9 | | 86.4 ± 16.3 | | 143.1 ± 18.8 | | 0.2 ± 0.2 | | 20.8 ± 2.8 | | 0.1 ± 0.1 | | 0.1 ± 0.1 | | 38.0 ± 4.4 | | 5.4 ± 1.5 | |
|  | P2 1pm | Av ± SD | 7.4 ± 5.0 | 596.0 ± 94.8 | 31.8 ± 3.4 | 7.8 ± 0.7 | | 191.7 ± 42.1 | | 110.4 ± 48.9 | | 78.9 ± 26.0 | | 139.5 ± 15.0 | | 0.2 ± 0.1 | | 20.6 ± 2.7 | | 0.1 ± 0.2 | | 0.1 ± 0.1 | | 37.3 ± 6.7 | | 5.4 ± 1.6 | |
|  | P5 6am | Av ± SD | 1.2 ± 1.3 | 603.5 ± 97.1 | 27.6 ± 4.5 | 7.6 ± 0.3 | | 164.2 ± 40.6 | | 87.3 ± 28.4 | | 69.6 ± 19.5 | | 123.75 ± 24.1 | | 0.1 ± 0.0 | | 12.7 ± 4.6 | | 0.4 ± 0.6 | | 0.2 ± 0.2 | | 29.4 ± 4.2 | | 5.6 ± 1.7 | |
|  | P5 1pm | Av ± SD | 10.4 ± 5.7 | 594.8 ± 96.7 | 30.9 ± 4.2 | 8.6 ± 0.7 | | 160.2 ± 33.8 | | 93.2 ± 26.0 | | 69.4 ± 25.6 | | 119.2 ± 20.8 | | 0.3 ± 0.2 | | 12.4 ± 4.1 | | 0.7 ± 1.1 | | 0.2 ± 0.3 | | 29.9 ± 5.2 | | 5.5 ± 1.7 | |

**WSP N-cycling gene expression (cDNA)**

**Supplementary Figure 2.** Heatmap of *BamoA, AamoA, nxrB*, *nrfA*, *nosZ, hzsA* and *nifH* FGA cDNA in Ponds 1, 2 and 5. For clarity, a subset of: 7 (99) *BamoA*; 6 (60) *AamoA*; 8 (21) *nxrB*; 5 (138) *nrfA*; 47 (182) *nosZ*; 8 (42) *hzsA*; and 55 (144) *nifH* probes are shown. A value of 100 means the signal was equal to that of the control probe (hyaBP60), whereas a value of 10 indicates that the signal was 10 % of the control. Colour coding is indicated on the colour bar on top of heatmap. N = 40. See Supplementary Table 2 for gene label and taxa identification details and the FGA data_cDNA supplementary excel for results values.

**References**

Francis, C.A., Roberts, K.J., Beman, J.M., Santoro, A.E., Oakley, B.B., 2005. Ubiquity and diversity of ammonia-oxidizing archaea in water columns and sediments of the ocean. Proc. Natl. Acad. Sci. U. S. A. 102, 14683–14688.

Harhangi, H.R., Roy, M.L., Alen, T. van, Hu, B., Groen, J., Kartal, B., Tringe, S.G., Quan, Z.-X., Jetten, M.S.M., Camp, H.J.M.O. den, 2012. Hydrazine Synthase, a Unique Phylomarker with Which To Study the Presence and Biodiversity of Anammox Bacteria. Appl Env. Microbiol 78, 752–758. https://doi.org/10.1128/AEM.07113-11

Kloos, K., Mergel, A., Rösch, C., Bothe, H., 2001. Denitrification within the genus Azospirillum and other associative bacteria. Funct. Plant Biol. 28, 991–998. https://doi.org/10.1071/pp01071

Mohan, S.B., Schmid, M., Jetten, M., Cole, J., 2004. Detection and widespread distribution of the nrfA gene encoding nitrite reduction to ammonia, a short circuit in the biological nitrogen cycle that competes with denitrification. FEMS Microbiol. Ecol. 49, 433–443. https://doi.org/10.1016/j.femsec.2004.04.012

Rotthauwe, J.-H., Witzel, K.-P., Liesack, W., 1997. The ammonia monooxygenase structural gene amoA as a functional marker: molecular fine-scale analysis of natural ammonia-oxidizing populations. Appl. Environ. Microbiol. 63, 4704–4712.

Throbäck, I.N., Enwall, K., Jarvis, A., Hallin, S., 2004. Reassessing PCR primers targeting nirS, nirK and nosZ genes for community surveys of denitrifying bacteria with DGGE. FEMS Microbiol. Ecol. 49, 401–417. https://doi.org/10.1016/j.femsec.2004.04.011

Vanparys, B., Spieck, E., Heylen, K., Wittebolle, L., Geets, J., Boon, N., De Vos, P., 2007. The phylogeny of the genus Nitrobacter based on comparative rep-PCR, 16S rRNA and nitrite oxidoreductase gene sequence analysis. Syst. Appl. Microbiol. 30, 297–308. https://doi.org/10.1016/j.syapm.2006.11.006

Zehr, J.P., McReynolds, L.A., 1989. Use of degenerate oligonucleotides for amplification of the nifH gene from the marine cyanobacterium Trichodesmium thiebautii. Appl. Environ. Microbiol. 55, 2522–2526.
